# In Response

**DOI:** 10.4269/ajtmh.2012.12-0277b

**Published:** 2012-11-07

**Authors:** Michael Brisson, Paul Brisson

**Affiliations:** C Co 3-10 AVN, Mountain DUSTOFF; Task Force Phoenix; 10th Combat Aviation Brigade; 10th Mountain Division (Light); Fort Drum, NY; E-mail: mike.brisson@us.army.mil; Deputy Commander of Surgery; Fort Belvoir Community Hospital; Fort Belvoir, VA; Email: paul.brisson@us.army.mil

Dear Sir:

Thank you for your interest in our manuscript. We too were surprised by the data that supported improved compliance with daily chemoprophylaxis when compared with weekly dosing. As you noted, conventional wisdom and historical evidence supports the conclusion that individuals would be more compliant with weekly medications than those that are required to be taken daily. We did not comment on that because we were not sure of the reason for inferior compliance with weekly dosing, but here is our speculation:

Although our numbers are small, we think poor compliance with once weekly dosing has many of the same etiological factors as the daily dosing: forgot, gastrointestinal side effects, not visualizing mosquitoes, winter months, busy operational tempo, with the possible additional significant factor that there has been an increase in the awareness by military service members of suicide and other psychiatric disturbances.

Our study was limited by convenience sampling. The distribution of the survey was accomplished using an e-mail address database of servicemembers assigned to a specific region of Afghanistan. We noted that this region had a particularly high number of flight crews and aviators. According to the U.S. Army Aeromedical Policy Letters,^1^ servicemembers are not permitted to remain on flight status while using mefloquine. Currently, the only approved medications for aviators and aircrew members include chloroquine phosphate, primaquine phosphate, and doxycycline.^1^

The term “aircrew members” is used to identify any individual requiring a flight physical to perform their crew duties. This includes the pilots, crew chiefs, flight engineers, and flight medics. Therefore, we speculate that our sample size was particularly limited because of the Army's medical standards for aviation service.

Thanks very much for your interest in our manuscript.
